# Electrophysiological and computational analysis of Ca_v_3.2 channel variants associated with familial trigeminal neuralgia

**DOI:** 10.1186/s13041-022-00978-9

**Published:** 2022-11-17

**Authors:** Emilio R. Mustafá, Eder Gambeta, Robin N. Stringer, Ivana A. Souza, Gerald W. Zamponi, Norbert Weiss

**Affiliations:** 1 Department of Pathophysiology, Third Faculty of Medicine, Charles University, Prague, Czech Republic; 2 Department of Clinical Neurosciences, Alberta Children’s Hospital Research Institute, Hotchkiss Brain Institute, Cumming School of Medicine, University of Calgary, Calgary, Canada; 3 Institute of Organic Chemistry and Biochemistry, Czech Academy of Sciences, Prague, Czech Republic

**Keywords:** Trigeminal neuralgia, Ion channel, Calcium channel, *CACNA1H*, Ca_v_3.2 channel, Channelopathy

## Abstract

Trigeminal neuralgia (TN) is a rare form of chronic neuropathic pain characterized by spontaneous or elicited paroxysms of electric shock-like or stabbing pain in a region of the face. While most cases occur in a sporadic manner and are accompanied by intracranial vascular compression of the trigeminal nerve root, alteration of ion channels has emerged as a potential exacerbating factor. Recently, whole exome sequencing analysis of familial TN patients identified 19 rare variants in the gene *CACNA1H* encoding for Ca_v_3.2T-type calcium channels. An initial analysis of 4 of these variants pointed to a pathogenic role. In this study, we assessed the electrophysiological properties of 13 additional TN-associated Ca_v_3.2 variants expressed in tsA-201 cells. Our data indicate that 6 out of the 13 variants analyzed display alteration of their gating properties as evidenced by a hyperpolarizing shift of their voltage dependence of activation and/or inactivation resulting in an enhanced window current supported by Ca_v_3.2 channels. An additional variant enhanced the recovery from inactivation. Simulation of neuronal electrical membrane potential using a computational model of reticular thalamic neuron suggests that TN-associated Ca_v_3.2 variants could enhance neuronal excitability. Altogether, the present study adds to the notion that ion channel polymorphisms could contribute to the etiology of some cases of TN and further support a role for Ca_v_3.2 channels.

## Introduction

Trigeminal neuralgia (TN) also referred as “tic douloureux” is a rare form of chronic neuropathic pain syndrome originating from the trigeminal nerve that supplies sensation to the face. TN is characterized by recurrent and chronic paroxysms of electric shock-like or stabbing pain in the orofacial region (for reviews see [1, 2]). The pain usually lasts from a few seconds to a few minutes and may be so intense that it triggers involuntary wincing, hence the term tic. Most cases of TN are sporadic but familial forms exist and are likely to be underestimated [3]. In both situations, the etiology of TN remains largely unknown and neurovascular compression of the trigeminal root nerve represents the primary theory for the underlying cause of the disease. However, the observation that many TN patients do not show any sign of neurovascular compression, and conversely that individuals with compression do not necessarily develop symptoms, suggested the existence of additional factors. Hence, an alteration of neuronal excitability resulting from abnormal functioning of ion channels has emerged as a potential underlying mechanism [4–7] and consistent with this notion, the sodium channel blockers carbamazepine and oxcarbazepine represent the first line therapy in TN [8]. Moreover, alterations of the expression of several ion channels including sodium, calcium, and potassium channels have been reported in TN patients [9] as well as in preclinical rodent models [10–17]. In addition, rare polymorphisms in ion channel genes were identified in TN patients [18–20] suggesting the existence of predisposing genetic factors and gain-of-function mutations (GoF) were reported for Na_v_1.6 [18], Ca_v_2.1 [21], TRPM7 [22, 23], and TRPM8 channels [24].

Recently, TN-associated polymorphisms in the gene *CACNA1H* encoding Ca_v_3.2 calcium channels were reported [25]. Ca_v_3.2 channels belong to the subfamily of low-voltage-activated T-type channels and are widely expressed throughout the nervous system where they play an essential role in the control of neuronal excitability [26]. Importantly, Ca_v_3.2 is expressed in all structures of the trigeminal pathway including trigeminal ganglion sensory neurons [27, 28], the spinal trigeminal nucleus (SpV) [17] as well as several thalamic nuclei such as the ventroposterior nucleus (VPM) [29] that receives projections from the SpV. Hence, Ca_v_3.2 channels may be of direct relevance for the transmission of trigeminal sensory information and a role for Ca_v_3.2 in TN-like syndrome was reported in a preclinical rodent model [17].

**Fig. 1:**
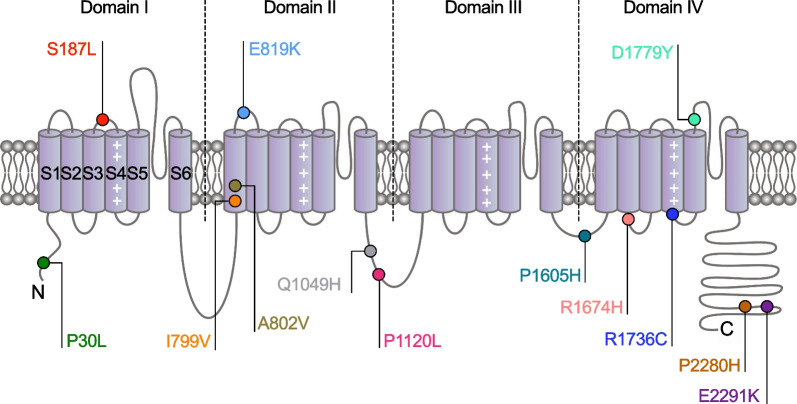
Schematic representation of the membrane topology of Ca_v_3.2 depicting the amino acid position of TN-associated variants characterized in this study

In this study, we aimed to provide a comprehension analysis of TN-associated *CACNA1H* variants with regard to their impact on the functioning of Ca_v_3.2 channels. Of the 19 variants reported [25], four had already been assessed for their impact on the biophysical properties of Ca_v_3.2 channels and revealed a variant-dependent effect such that G563R and P566T produced a GoF of the channel, E286K caused a mild loss-of-function (LoF), and H526Y did not cause any alteration [17, 30]. We now report the functional characterization of 13 additional variants. Seven of these variants are located within cytoplasmic regions of Ca_v_3.2 including the N-terminal region (P30L), the loop connecting domains II and III (Q1049H and P1120L), the loop connecting domains III and IV (P1605H), the linker connecting transmembrane segments S2-S3 of domain IV (R1674), and the C-terminal region (P2280H and E2291K). Four additional variants are mapped within important structural determinants of the channel including the transmembrane segment S1 of domain II (I799V and A802V), the end of the S4 voltage-sensor of domain IV (R1736C), and the fourth pore-forming loop (D1779Y). The two remaining variants are localized within the extracellular linkers connecting transmembrane segments S3-S4 of domain I (S187L) and S1-S2 of domain II (E819K) (Fig. 1). Electrophysiological analysis of recombinant TN-associated Ca_v_3.2 variants in tsA-201 cells revealed a significant alteration in the gating properties of 7 out of the 13 variants analyzed. In addition, introduction of these variants in a computational model of reticular thalamic neuron (nRT) enhanced rebound burst firing of action potentials. Taken together, these data suggest that altered gating of TN-associated Ca_v_3.2 variants may enhance neuronal excitability which could potentially contribute to the etiology of TN.

## Materials and methods

### Plasmid cDNA constructs and site-directed mutagenesis

The Ca_v_3.2 variants were generated by site directed mutagenesis performed by GenScript using the wild-type human Ca_v_3.2 (containing exon 26) in pcDNA3.1 (kindly provided by Dr. Terrance Snutch) as template. The fidelity of all constructs was confirmed by full-length sequencing of the coding region.

### Cell culture and heterologous expression

Human embryonic kidney tsA-201 cells were grown in DMEM medium supplemented with 10% fetal bovine serum and 1% penicillin/streptomycin (all media purchased from Invitrogen) and maintained under standard conditions at 37 °C in a humidified atmosphere containing 5% CO_2_. Heterologous expression was performed by transfecting cells with 5 μg of plasmid cDNAs encoding for Ca_v_3.2 variants and empty pEGFP vector as transfection marker using the calcium/phosphate method.

### Patch clamp electrophysiology

Patch clamp recordings of T-type currents in tsA-201 cells expressing Ca_v_3.2 variants were performed 72 h after transfection in the whole-cell configuration at room temperature (22–24 °C) in a bath solution containing (in millimolar): 10 BaCl_2_, 125 CsCl, 1 MgCl_2_, 10 D-glucose, 10 4-(2-hydroxyethyl)-1-piperazineethanesulfonic acid (HEPES) (pH 7.4 with CsOH). Patch pipettes were filled with a solution containing (in millimolar): 110 CsCl, 3 Mg-ATP, 0.5 Na-GTP, 2.5 MgCl_2_, 5 D-glucose, 10 EGTA, and 10 HEPES (pH 7.4 with CsOH), and had a resistance of 2–4MΩ. The calculated liquid junction potential was about − 2.6 mV and therefore was corrected from the recordings. Recordings were performed using an Axopatch 200B amplifier (Axon Instruments) and acquisition and analysis were performed using pClamp 10 and Clampfit 10 softwares, respectively (Axon Instruments). The linear leak component of the current was corrected using a P/4 subtraction protocol and current traces were digitized at 10 kHz and filtered at 2 kHz.

The voltage dependence of activation of Ca_v_3.2 channels was determined by measuring the peak of the T-type current in response to 140 ms depolarizing steps from − 80 mV to + 20 mV in 5 mV increments preceded by a 200 ms hyperpolarizing prepulse to − 110 mV from a holding membrane potential of − 100 mV. The current–voltage relationship (*I*/*V*) curve was fitted with the following modified Boltzmann Eq. (1):

1 $$I\left(V\right)= Gmax \frac{(V-V\mathrm{rev})}{1+ \mathrm{exp}\frac{(V0.5-V) }{k}}$$

with *I*(*V*) being the peak current amplitude at the command potential *V*, *G*_max_ the maximum conductance, *V*_rev_ the reversal potential, *V*_0.5_ the half-activation potential, and *k* the slope factor. The voltage dependence of the whole-cell T-type channel conductance was calculated using the following modified Boltzmann Eq. (2):

2 $$G\left(V\right)= \frac{Gmax}{1+ \mathrm{exp}\frac{(V0.5-V) }{k}}$$

with *G*(*V*) being the T-type channel conductance at the command potential *V*.

The voltage dependence of the steady-state inactivation of Ca_v_3.2 channels was determined by measuring the peak T-type current amplitude in response to a 50 ms depolarizing step to -30 mV applied after a 1 s-long conditioning prepulse ranging from -110 mV to -15 mV in 5 mV increments. The current amplitude obtained during each test pulse was normalized to the maximal current amplitude and plotted as a function of the prepulse potential. The voltage dependence of the steady-state inactivation was fitted with the following two-state Boltzmann function (3):

3 $$I\left(V\right)= \frac{Imax}{1+ \mathrm{exp}\frac{(V-V0.5) }{k}}$$

with *I*_max_ corresponding to the maximal peak current amplitude and *V*_0.5_ to the half-inactivation voltage.

The recovery from inactivation was assessed using a double-pulse protocol preceded by a 50 ms-long hyperpolarizing prepulse to -110 mV from a holding potential of -100 mV. The cell membrane was depolarized for 2 s at -20 mV (inactivating prepulse) to ensure complete inactivation of the channel, and then to -20 mV for 150 ms (test pulse) after an increasing time period (interpulse) ranging between 1 ms and 8 s at -110 mV. The peak current from the test pulse was plotted as a ratio of the maximum prepulse current versus interpulse interval. The data were fitted with the following single-exponential function (4):

4 $$\frac{I}{Imax}=A \times (1- exp\frac{-t}{\tau })$$

where τ is the time constant for channel recovery from inactivation.

### Computational modeling

Simulation of thalamic reticular neuron (nRT) firing was performed using the NEURON simulation environment (https://senselab.med.yale.edu/ModelDB/) [31] in the three-compartment model previously described [32]. The electrophysiological properties of wild-type and TG-associated Ca_v_3.2 variants obtained experimentally were modeled using Hodgkin-Huxley equations as previously described [33] and introduced into the model. To take into account the relative expression of Cav3.2 channels in nRT neurons (about 40% of Ca_v_3.2 and 60% of Ca_v_3.3 [34]) and the heterozygous nature of TN-associated Ca_v_3.2 variants, only 20% of the T-type channel conductance described in the original model was altered with experimental values obtained for WT and TN-associated Ca_v_3.2 variants. The simulation was performed at a holding potential set to -70 mV and the electrical membrane potential of the virtual soma was monitored in response to a 200ms-long hyperpolarizing and depolarizing current injection in order to assess rebound and tonic firing, respectively.

### Statistical analysis

Average data are presented as mean ± S.E.M. for *n* measurements. Statistical analysis was performed using GraphPad Prism 8. A Kolmogorov–Smirnov normality test was performed and statistical significance was assessed using Kruskal–Wallis test with Dunn’s post-test. Datasets were considered significantly different for p ≤ 0.05.

## Results

### ***Expression of TN-associated Ca***_***v***_***3.2 variants***

**Fig. 2:**
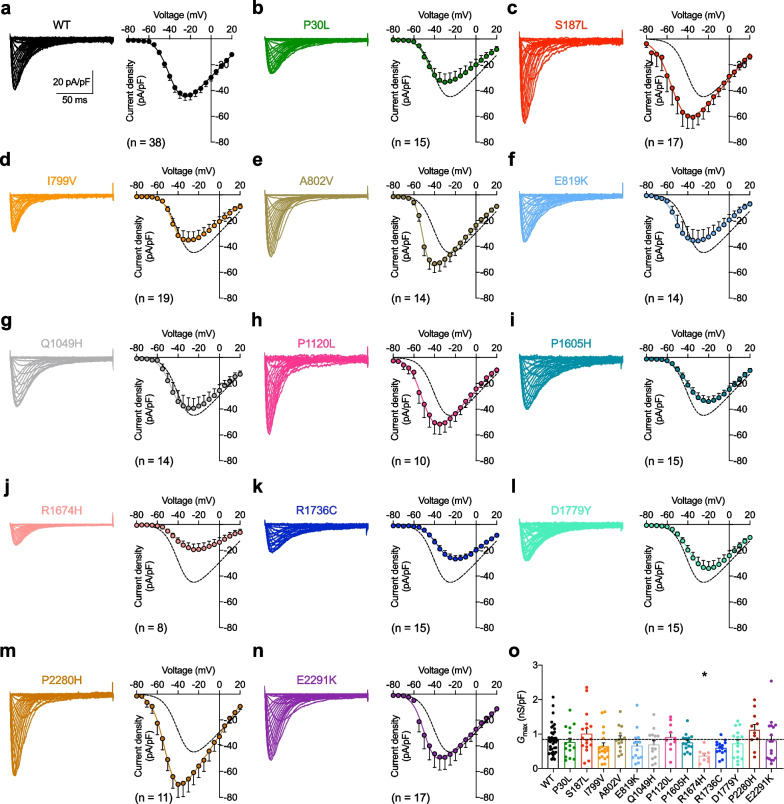
Expression of TN-associated Ca_v_3.2 variants. **a** Representative whole-cell T-type current traces recorded in tsA-201 cells expressing Ca_v_3.2 wild-type (WT) in response to 140 ms depolarizing steps to values ranging between − 80 mV and + 20 mV (left panel). Corresponding mean current–voltage relationship (*I*/*V*) (right panel) fitted with the modified Boltzmann function (1). **b-n** Legend same as (**a**) but for cells expressing TN-associated Ca_v_3.2 variants. The dashed line depicts the *I*/*V* curve of the WT channel for comparison. **o** Corresponding mean maximal macroscopic conductance (*G*_max_) values obtained from the fit of the *I*/*V* curves

To assess the functional impact of TN-associated *CACNA1H* variants, tsA-201 cells were transiently transfected with plasmids encoding human Ca_v_3.2 wild-type (WT) and TN-associated variants for electrophysiological analysis. Whole-cell patch clamp recordings in tsA-201 cells expressing wild-type (WT) and TN-associated Ca_v_3.2 variants revealed that all variants were functionally expressed and generated a characteristic low-voltage-activated T-type current similar to WT channels (Fig. 2a–n, left panels). The maximal whole-cell macroscopic T-type channel conductance (*G*_max_) obtained from the fit of the current–voltage relationships (Fig. 2a–n, right panels) revealed no significant difference between cells expressing Ca_v_3.2 variants compared to cells expressing the WT channel except for the R1674H variant were *G*_max_ was reduced by 56% (*p* = 0.0185) (Fig. 2o and Table 1).

**Table 1:** Steady-state activation properties of TN-associated human Ca_v_3.2 variants expressed in tsA-201 cells

	Activation						
Ca_V_3.2	*G*_max_ (nS/pF)	*p*	*V*_0.5_ (mV)	*p*	*k* (mV)	*p*	(n)
WT	0.84 ± 0.07		− 38.17 ± 0.80		6.38 ± 0.35		38
P30L	0.77 ± 0.11	> 0.9999	− 44.18 ± 1.24	0.1251	4.70 ± 0.51	0.2024	15
S187L	1.01 ± 0.15	> 0.9999	− 51.63 ± 2.56	< 0.0001	3.47 ± 0.50	0.0006	17
I799V	0.65 ± 0.10	0.6889	− 41.78 ± 1.58	0.5519	5.62 ± 0.69	> 0.9999	19
A802V	0.85 ± 0.09	> 0.9999	− 48.82 ± 1.14	< 0.0001	3.56 ± 0.45	0.0015	14
E819K	0.66 ± 0.12	> 0.9999	− 48.19 ± 2.97	0.0208	4.31 ± 0.53	0.0491	14
Q1049H	0.70 ± 0.11	> 0.9999	− 41.88 ± 2.42	> 0.9999	4.99 ± 0.67	0.7660	14
P1120L	0.91 ± 0.13	> 0.9999	− 49.55 ± 3.17	0.0106	4.23 ± 0.56	0.1175	10
P1605H	0.76 ± 0.06	> 0.9999	− 34.53 ± 1.60	> 0.9999	8.17 ± 0.34	0.2336	15
R1674H	0.37 ± 0.07	0.0185	− 38.23 ± 2.30	> 0.9999	6.49 ± 0.99	> 0.9999	8
R1736C	0.60 ± 0.05	0.8669	− 32.42 ± 1.38	0.4164	7.39 ± 0.29	> 0.9999	15
D1779Y	0.72 ± 0.11	> 0.9999	− 33.86 ± 1.12	> 0.9999	8.08 ± 0.27	0.2457	15
P2280H	1.12 ± 0.16	> 0.9999	− 53.12 ± 2.31	< 0.0001	3.87 ± 0.37	0.0159	11
E2291K	0.81 ± 0.15	> 0.9999	− 46.13 ± 2.01	0.0268	4.91 ± 0.69	0.1754	17

### ***TN-associated CACNA1H variants alter the gating properties of Ca***_***v***_***3.2 channels***

**Fig. 3:**
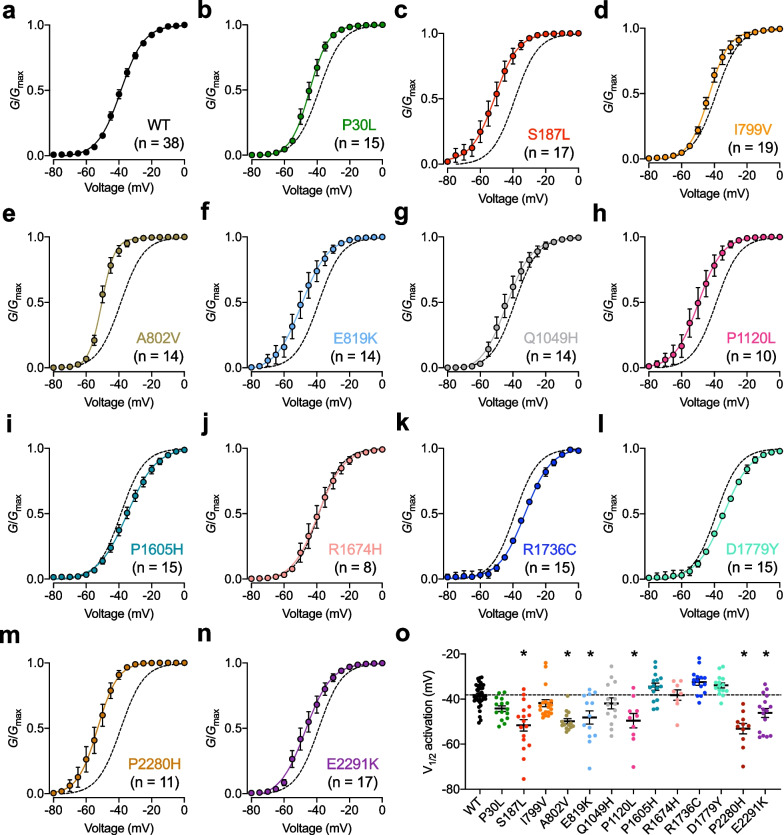
Activation properties of TN-associated Ca_v_3.2 variants. **a** Mean normalized voltage dependence of activation of wild-type (WT) Ca_v_3.2 channels fitted with the modified Boltzmann function (2). **b**–**n** Legend same as (**a**) but for TN-associated Ca_v_3.2 variants. The dashed line depicts the activation curve of the WT channel for comparison. **o** Corresponding mean half-activation potential (*V*_1/2_ activation) values obtained from the fit of the conductance curve

**Fig. 4:**
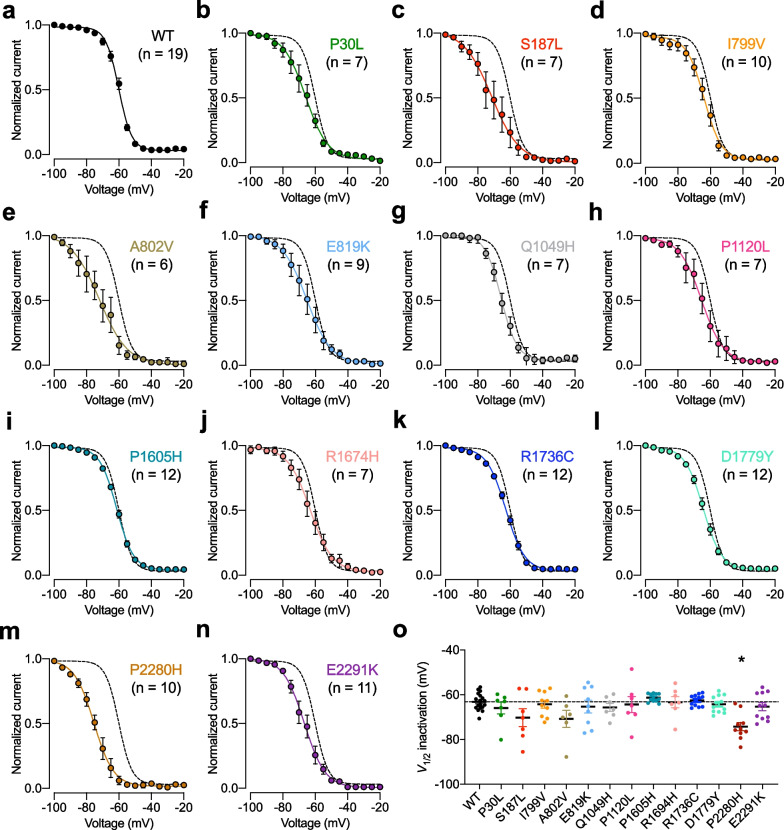
Inactivation properties of TN-associated Ca_v_3.2 variants. **a** Mean normalized voltage dependence of steady-state inactivation of wild-type (WT) Ca_v_3.2 channels fitted with the two-state Boltzmann function (3). **b**–**n** Legend same as **a** but for TN-associated Ca_v_3.2 variants. The dashed line depicts the inactivation curve of the WT channel for comparison. **o** Corresponding mean half-inactivation potential (*V*_1/2_ inactivation) values obtained from the fit of the inactivation curve

**Fig. 5:**
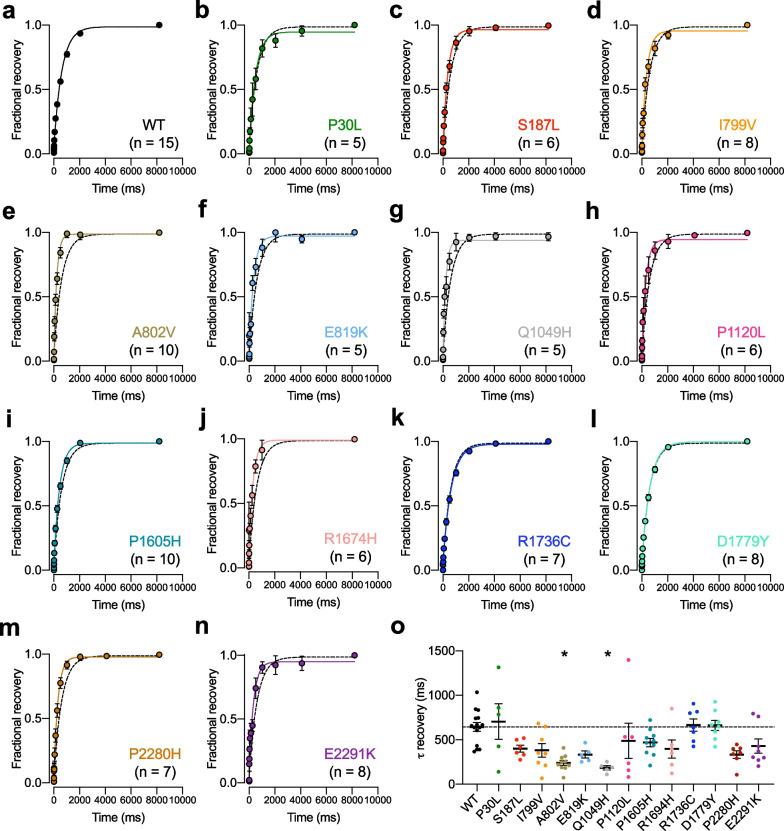
Recovery from inactivation properties of TN-associated Ca_v_3.2 variants. **a** Mean normalized recovery from inactivation kinetic of wild-type (WT) Ca_v_3.2 channels fitted with the single-exponential function (4). **b**–**n** Legend same as (**a**) but for TN-associated Ca_v_3.2 variants. The dashed line depicts the recovery from inactivation of the WT channel for comparison. **o** Corresponding mean time constant τ values of recovery obtained from the fit of the recovery from inactivation curve

Next, we aimed to assess the gating properties of TN-associated Ca_v_3.2 variants. First, we analyzed the voltage dependence of activation of the channels. In 6 (S187L, A802V, E819K, P1120L, P2280H, and E2291K) out of the 13 variants analyzed, the mean half-activation potential of the T-type current was significantly shifted toward more hyperpolarized potentials by − 8.0 mV (E2291K, *p* = 0.0268) up to − 15.0 mV (P2280H, p < 0.0001) relative to WT channels which is consistent with a GoF of the channels (Fig. 3a–o and Table 1). In addition, a significant decrease of the activation slope factor (*k*) was observed for S187L, A802V, E819K, and P2280H variants suggesting an increased coupling between the channel voltage-sensor and the pore opening again consistent with a GoF which may be particularly relevant for voltage changes close to the resting membrane potential where first openings of the channel occur (Table 1). To gain additional insights into the electrophysiological properties of TN-associated Ca_v_3.2 variants, we then assessed their voltage dependence of inactivation. A statistically significant hyperpolarizing shift of the voltage dependence of inactivation by -12.6 mV (*p* = 0.0015) relative to WT channels was observed for the P2280H variant and a similar trend albeit not statistically significant was observed for S187L (− 8.6 mV, *p* = 0.8716) and A802V variants (− 9.2 mV, *p* = 0.3699) whereas the remaining variants remained unaltered (Fig. 4a–o and Table 2). The alteration of the voltage dependence of inactivation is consistent with a LoF of the channel variants although the extent to which it may affect channel activity will largely depend on the resting membrane potential of cells, with a more pronounced effect in cells with a comparatively depolarized resting potential. In contrast, the kinetics of recovery from inactivation were accelerated by 2.7-fold for A802V (*p* = 0.0001) and by 3.5-fold for Q1049H variants (*p* = 0.0005) compared to WT channels, and a similar trend (albeit not statistically significant) was observed for several other variants indicative of a GoF (Fig. 5a–o and Table 2).

**Table 2:** Steady-state inactivation and recovery from inactivation properties of TN-associated human Ca_v_3.2 variants expressed in tsA-201 cells

	Inactivation					Recovery from inactivation		
Ca_V_3.2	*V*_0.5_ (mV)	*p*	*k* (mV)	*p*	(n)	τ (ms)	*p*	(n)
WT	− 61.61 ± 1.75		− 4.48 ± 0.22		19	644 ± 49		15
P30L	− 65.95 ± 2.67	> 0.9999	− 4.75 ± 0.44	> 0.9999	7	705 ± 200	> 0.9999	5
S187L	− 70.21 ± 3.99	0.8716	− 4.10 ± 0.34	> 0.9999	7	400 ± 39	0.6749	6
I799V	− 64.22 ± 1.71	> 0.9999	− 4.27 ± 0.51	> 0.9999	10	382 ± 76	0.1542	8
A802V	− 70.79 ± 3.80	0.3699	− 4.16 ± 0.39	> 0.9999	6	241 ± 28	0.0001	10
E819K	− 65.31 ± 2.86	> 0.9999	− 5.18 ± 0.55	> 0.9999	9	335 ± 38	0.1564	5
Q1049H	− 65.68 ± 1.74	> 0.9999	− 4.44 ± 0.28	> 0.9999	7	185 ± 24	0.0005	5
P1120L	− 64.38 ± 3.53	> 0.9999	− 3.64 ± 0.57	> 0.9999	7	489 ± 197	0.4634	6
P1605H	− 61.32 ± 0.51	> 0.9999	− 5.10 ± 0.19	> 0.9999	12	470 ± 46	> 0.9999	10
R1674H	− 63.29 ± 2.49	> 0.9999	− 6.05 ± 1.04	> 0.9999	7	397 ± 103	0.3918	6
R1736C	− 62.48 ± 0.73	> 0.9999	− 5.50 ± 0.11	0.0191	12	665 ± 68	> 0.9999	7
D1779Y	− 64.23 ± 1.07	> 0.9999	− 5.03 ± 0.11	> 0.9999	12	662 ± 58	> 0.9999	8
P2280H	− 74.22 ± 1.89	0.0015	− 4.56 ± 0.37	> 0.9999	10	334 ± 42	0.0724	7
E2291K	− 65.27 ± 1.83	> 0.9999	− 4.23 ± 0.19	> 0.9999	11	429 ± 80	0.3967	8

### ***Ca***_***v***_***3.2-dependent window current is altered by TN-associated CACNA1H variants***

**Fig. 6:**
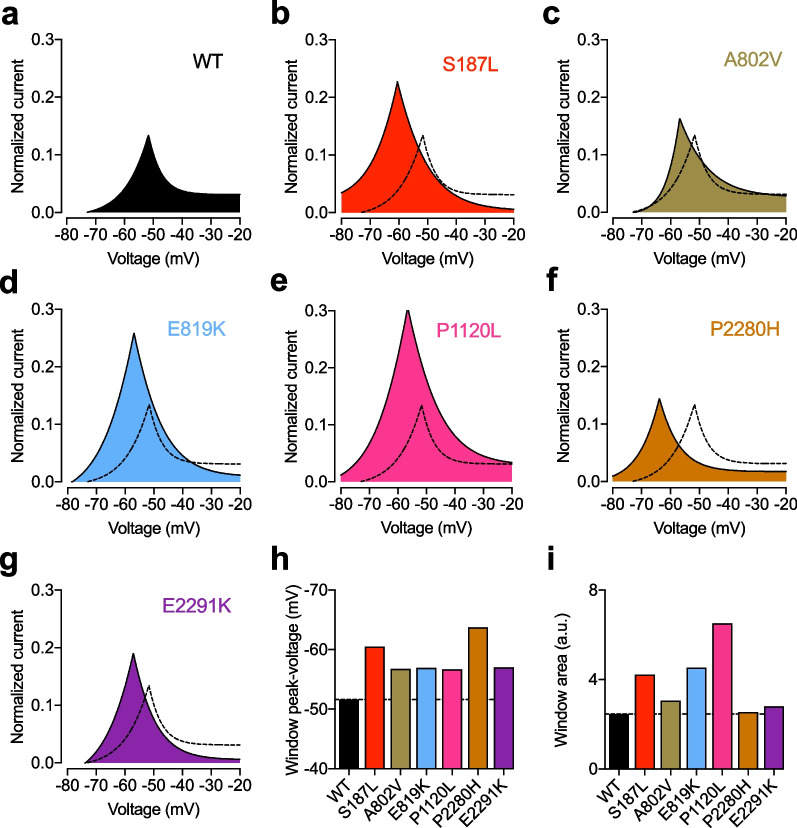
Window current of TN-associated Ca_v_3.2 variants. **a** Window current of wild-type (WT) Ca_v_3.2 illustrated by the overlap of the mean activation and inactivation curves. **b-g** Legend same as (**a**) but for TN-associated Ca_v_3.2 variants. The dashed line depicts the window current of the WT channel for comparison. **h** Corresponding peak-voltage values of the window current: WT (− 51.6 mV), S187L (− 60.5 mV), A802V (− 56.8 mV), E819K (− 56.9 mV), P1120L (− 56.7 mV), P2280H (− 63.8 mV), E2291K (− 57.0 mV). **i** Corresponding magnitude values of the window current measured as the area under the curve: WT (2.5 a.u.), S187L (4.2 a.u.), A802V (3.1 a.u.), E819K (4.5 a.u.), P1120L (6.5 a.u.), P2280H (2.5 a.u.), E2291K (2.8 a.u.)

Because several TN-associated Ca_v_3.2 variants showed alterations in the voltage dependence of activation and/or inactivation, we aimed to assess the impact on the T-type window current by visualizing the overlapping area between the activation and inactivation curves (Fig. 6a–g). In all TN-associated Ca_v_3.2 variants for which the voltage dependence of activation and/or inactivation was altered, the window current was displaced toward more hyperpolarized potentials with the peak-voltage shifted by − 5 mV (P1120L) up-to − 12 mV (P2280H) (Fig. 6h). This effect was accompanied by an increased magnitude of the window current (except for the P2280H variant) ranging from 14% (E2291K) up-to 165% increase (P1120L) (Fig. 6i).

### ***TN-associated Ca***_***v***_***3.2 variants increase neuronal firing in a computational model of thalamic neurons***

**Fig. 7:**
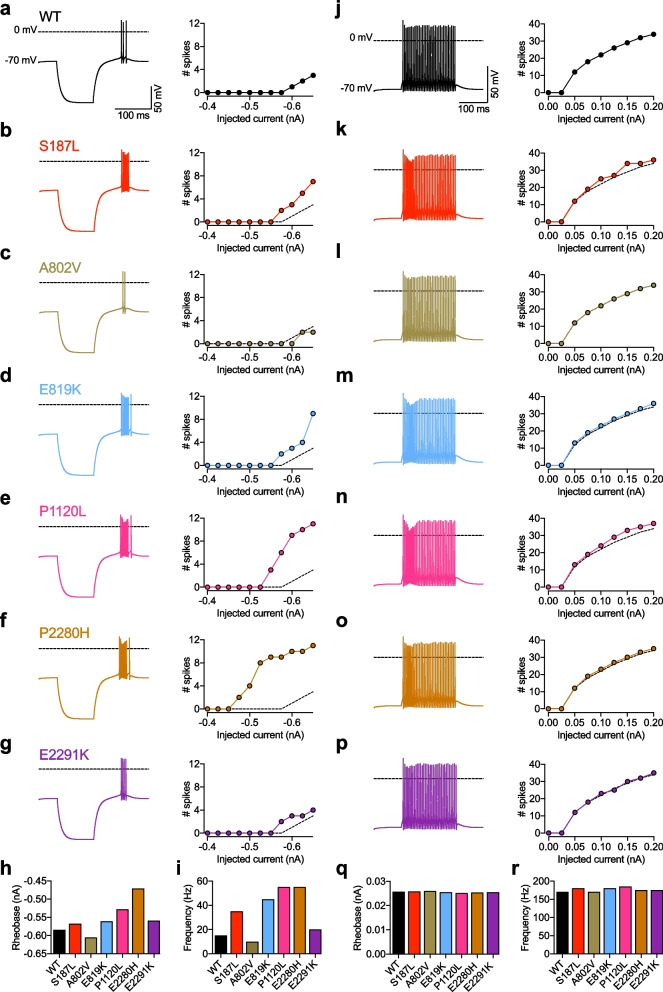
Computer simulation of nRT neuron firing. **a** Representative electrical membrane potential of the virtual soma containing wild-type (WT) Ca_v_3.2 channels in response to a 200 ms-long hyperpolarizing current injection of − 0.65 nA (left panel) and corresponding number of spikes during the rebound as a function of the current injected (right panel). **b**–**g** Legend same as **a** but for TN-associated Ca_v_3.2 variants. The dashed line depicts the number of spikes for WT channels for comparison. **h** Minimum current injection (rheobase) necessary to trigger rebound action potentials: WT (− 0.585 nA), S187L (− 0.568 nA), A802V (− 0.606 nA), E819K (− 0.561 nA), P1120L (− 0.528 nA), E2280H (− 0.471 nA), and E2291K (− 0.559 nA). **i** Rebound firing frequency at -0.65 nA current injection: WT (15 Hz), S187L (35 Hz), A802V (10 Hz), E819K (45 Hz), P1120L (55 Hz), E2280H (55 Hz), and E2291K (20 Hz). **J**–**p** Legend same as **a**–**g** but for depolarizing current injections. Representative membrane potentials are shown in response to 0.2 nA current injection. **q** Minimum current injection (rheobase) necessary to trigger tonic action potentials: WT (0.0257 nA), S187L (0.0258 nA), A802V (0.0260 nA), E819K (0.0255 nA), P1120L (0.0251 nA), E2280H (0.0254 nA), and E2291K (0.0255 nA). **r** Action potential frequency at 0.2 nA current injection: WT (170 Hz), S187L (180 Hz), A802V (170 Hz), E819K (180 Hz), P1120L (185 Hz), E2280H (175 Hz), and E2291K (175 Hz)

Given that Ca_v_3.2 channels are highly expressed in thalamic neurons where they play an essential role in regulating neuronal excitability [35] and considering that the thalamus is a key relay station in the trigeminal sensory pathway [36], we aimed to simulate the functional consequence of TN-associated Ca_v_3.2 variants on neuronal electrical activities using a computational model of reticular thalamic neuron (nRT). The simulation was performed with Ca_v_3.2 variants for which an alteration of the voltage dependence of activation and/or inactivation was observed and the original model was altered in order to account for the relative contribution of Ca_v_3.2 channels to the overall native T-type conductance and also to account for the heterozygous nature of TN-associated Ca_v_3.2 variants (see *Methods*). Simulation of the neuronal membrane potential showed that hyperpolarizing current injections triggered rebound burst firing with WT as well as with TN-associated Ca_v_3.2 variants (Fig. 7a–g). However, the minimum current necessary to trigger rebound firing (rheobase) was significantly less for TN-associated Ca_v_3.2 variants (except A802V) compared to WT channels (Fig. 7h). Moreover, the firing frequency was increased (Fig. 7i). In contrast, when the firing was triggered with depolarizing current injections there was no major effect between WT and TN-associated Ca_v_3.2 variants (Fig. 7j–r).

## Discussion

Polymorphisms in the *CACNA1H* gene have been reported in a number of human disorders [37] and GoF mutations in Ca_v_3.2 are linked to primary aldosteronism (PA) [38–40] and idiopathic generalized epilepsy (IGE) [41]. In contrast, LoF mutations were documented in autism spectrum disorders [42], neuromuscular disorders [43–45], and developmental and epileptic encephalopathy [46].

In this study, we report the functional characterization of 13 Ca_v_3.2 missense variants identified in TN patients. Patch clamp recordings of T-type currents in tsA-201 cells expressing recombinant TN-associated Ca_v_3.2 variants showed that all variants were functional with no significant alteration in their maximal macroscopic conductance except for the R1674H variant for which the conductance was reduced. This effect was not further investigated but may have been caused by a decreased trafficking of the channel to the plasma membrane and/or decreased stability. In contrast, of the 13 Ca_v_3.2 variants analyzed, 6 variants (S187L, A802V, E819K, P1120L, P2280H, and E2291K) displayed alterations in their gating properties evidenced by a recurrent hyperpolarized shift of the voltage dependence of activation consistent with a GoF of the channels. An additional acceleration of the recovery from inactivation was also observed for A802V and Q1049H. Although these variants showed similar alterations in their gating properties, they did not segregate into a particular region of Ca_v_3.2. Nonetheless, some of the channel molecular determinants containing TN-associated variants are known to contribute to the gating of Ca_v_3.2. For instance, the II-III loop containing variant P1120L and the C-terminus containing variants P2280H and E2291K were previously reported to affect the voltage dependence of T-type channels [47–49]. Moreover, the GoF effect of TN-associated variants in the C-terminus of Ca_v_3.2 is reminiscent of what was reported for several variants associated with (IGE) and PA [40, 50]. Importantly, when introduced into a computational model of nRT neuron, the 6 variants reduced the threshold for rebound burst firing implying an overall GoF effect. This is consistent with previous findings in various types of neurons showing that upregulation of T-type channel activity underlies reduced threshold for rebound burst firing [51–55]. While our modeling was performed in a computational model of nRT neurons, it may anticipate some of the possible effects of TN-associated Ca_v_3.2 variants on the functioning of the trigeminal pathway for several reasons. First, although T-type dependent rebound burst firing has yet to be shown in trigeminal ganglion (TG) sensory neurons, it has been documented in dorsal root ganglion sensory neurons [56] and it is a possibility that it does also occur in TG neurons. Second, a low-threshold calcium conductance (presumably mediated by T-type channels) leading to calcium spikes and rebound burst firing has been reported in neurons of the brain stem trigeminal nuclei [57]. Third, the trigeminal pathway gates through the thalamus in particular via the VPM where T-type channels contribute to rebound burst firing [58]. And finally, alteration of thalamocortical rhythmic activities mediated by T-type channels has been implicated in the development of trigeminal pain [14]. Hence, all of these aspects suggest that alteration of rebound burst firing caused by TN-associated Ca_v_3.2 variants could potentially contribute to the sensitization of the trigeminal pathway. In addition, alteration of the channel gating properties resulted in a hyperpolarizing displacement of the voltage dependence of the window current which implies an increased passive influx of calcium around the resting membrane potential of cells. Considering that the voltage range of the window current is an important determinant of neuronal electrical activities and calcium oscillations [59], this may further contribute to enhance neuronal activity. These data are consistent with a previous report showing that re-expression of a GoF TN-associated Ca_v_3.2 variant in cultured TG neuron increased neuronal excitability [17]. The question then remains as to why TN patients harboring GoF Ca_v_3.2 variants did not show signs of IGE or PA. It is a possibility that the gating alterations caused by these variants and affecting only the rebound burst firing in the absence of general alteration of the tonic firing is not enough to cause additional disease phenotypes.

In conclusion, our functional analysis of 13 Ca_v_3.2 variants identified in TN patients revealed an overall GoF of the channel for 7 of these variants that could potentially contribute to the sensitization of the trigeminal pathway. Although these gating effects are reminiscent of what was previously reported for TN-associated variants in Na_v_1.6 [18], Ca_v_2.1 [21], TRPM7 [22, 23], and TRPM8 channels [24], it is important to consider that our functional analysis in a heterologous expression system provides only a snapshot of the phenotype of a mutation. Hence, additional analysis of these variants in native conditions will be necessary to further validate these findings. Moreover, it is a possibility that the variants for which we did not observe any gating alteration will show different phenotypes in a more complex physiological environment. Finally, although most of the gating alterations were in general consistent with a GoF of the channel, it is important to consider that one variant was associated with a LoF suggesting that GoF phenotypes in ion channels may not represent a universal feature in TN. For instance, the expression level of *SCN9A* (Na_v_1.7) and *SCN10A* (Na_v_1.8) is reduced in gingival tissue of TN patients [9], as well as in preclinical models of TN [11] implying a LoF phenotype, although this mechanism may occur as a protective mechanism to normalize neuronal excitability. Nonetheless, it is striking to note that all patients exhibiting idiopathic TN with concomitant continuous pain (iTN-2) harbored GoF Ca_v_3.2 variants. In contrast, all Ca_v_3.2 variants identified in patients with congenital TN and concomitant pain (cTN-2) did not cause any alteration of the channel (Table 3). While additional studies are necessary to assess the exact role of Ca_v_3.2 in the processing of trigeminal sensory information, our data add to the notion that rare *CACNA1H* variants may contribute to the etiology of TN. In that respect, the T-type channel blocker valproic acid was shown to be effective to mitigate pain in some TN patients [60] suggesting that other antiepileptic T-type channel blockers ethosuximide, zonizamide, and nimodipine [26] should also be considered especially in patients resistant to first line therapies.

**Table 3:** Summary of gating effects of TN-associated Ca_v_3.2 variants in relation to the clinical phenotype of patients

Idiopathic TN**	Ca_v_3.2 variant	Classical TN**	Ca_v_3.2 variant
iTN-1	E286K*	cTN-1	*E2291K*
iTN-1	G563R*		
iTN-1	**R1674H**	cTN-2	***P30L***
iTN-1	***D1779Y***	cTN-2	H526Y*
		cTN-2	***I799V***
iTN-2	P566T*	cTN-2	***P1605H***
iTN-2	*E819K*	cTN-2	***R1736C***
iTN-2	*Q1049H*	cTN-2	***P30L***
iTN-2	*P1120L*		
iTN-2	*P2280H*		

Italic: GoF

Bold: LoF

Bolditalic: Neutral

*iTN* idiopathic trigeminal neuralgia, *cTN* classical trigeminal neuralgia; (− 1), purely paroxysmal; (− 2) with concomitant continuous pain. *According to [17]. **According to [25]. Two GoF variants (S187L and A802V) are not included in this table since their clinical phenotype was not fully defined (atypical facial pain and TN without further information, respectively)

## Data Availability

All data generated or analyzed during this study are included in this published article and its supplementary information files.

## Abbreviations

cTN-1: Classical trigeminal neuralgia purely paroxysmal
cTN-2: Classical trigeminal neuralgia with concomitant continuous pain
GoF: Gain-of-function
IGE: Idiopathic generalized epilepsy
iTN-1: Idiopathic trigeminal neuralgia purely paroxysmal
iTN-2: Idiopathic trigeminal neuralgia with concomitant continuous pain
LoF: Loss-of-function
nRT: Reticular thalamic neuron
PA: Primary aldosteronism
SpV: Spinal trigeminal nucleus
TG: Trigeminal ganglion
TN: Trigeminal neuralgia
VPM: Ventroposterior nucleus
WT: Wild-type
